# Neuroprotective effect of omega‐3 fatty acids on spinal cord injury induced rats

**DOI:** 10.1002/brb3.1339

**Published:** 2019-06-21

**Authors:** Jiaqi Bi, Chong Chen, Peiyu Sun, Haining Tan, Fan Feng, Jianxiong Shen

**Affiliations:** ^1^ Department of Orthopedic Surgery Peking Union Medical College Hospital, Chinese Academy of Medical Sciences and Peking Union Medical College Beijing China; ^2^ Department of Orthopedic Surgery The First Hospital of Harbin City Harbin Heilongjiang China; ^3^ Department of Orthopedics Beijing Hospital of Traditional Chinese Medicine Beijing China; ^4^ Department of Orthopedics Surgery, Renji Hospital, School of Medicine Shanghai Jiao Tong University Shanghai China

**Keywords:** apoptosis, inflammation, omega‐3 fatty acid, oxidative stress, rat

## Abstract

**Introduction:**

In this study, the effects of omega‐3 fatty acids were examined in a rat model of spinal cord injury.

**Methods:**

The rats were classified into sham, control, spinal cord injury plus 50 mg/kg Omega‐3 fatty acids and spinal cord injury plus 100 mg/kg Omega‐3 fatty acids. The levels of oxidative, apoptotic, and inflammatory markers were examined in each of these groups.

**Results:**

Altered lipid peroxidation, reduced glutathione (GSH), superoxide dismutase (SOD), glutathione peroxidase (Gpx), and catalase were normalized. Omega‐3 fatty acid supplementation decreased tumor necrosis factor‐alpha (TNF‐α) and interleukin‐6 (IL‐6) levels by >50%. TNF‐α and IL‐6 mRNA expression were reduced. Caspase‐3, p53, bax, and pro‐NGF mRNA expression levels were increased by 1.3‐, 1.4‐, 1.2‐, and 0.9‐fold, respectively, whereas bcl‐2 mRNA expression was decreased by 0.77‐fold in control rats. Omega‐3 fatty acid supplementation decreased p53, caspase‐3, bax, and pro‐NGF mRNA expression by >40%, while the level of bcl‐2 mRNA expression was increased by 286.9%. Omega‐3 fatty acid supplementation decreased caspase‐3 and p53 protein expression by >30%.

**Conclusion:**

Taken together, our results suggested that omega‐3 fatty acid supplementation reduced oxidative stress, apoptosis, and the levels of inflammatory markers in ischemia‐reperfusion‐induced rats.

## 1 INTRODUCTION

Omega‐3 fatty acids are important polyunsaturated fatty acids with some roles in normal cellular metabolism (Scorletti & Byrne, 2013). Essential fatty acids have been reported to be important in membrane fluidity, inflammatory eicosanoids, and neural membrane oxidation (Salvati, Attorri, DiBenedetto, DiBiase, & Leonardi, 2006). The daily recommended level of omega‐3 fatty acid is 300 mg for healthy adults. Leukotriene‐5, thromboxane‐3, and prostaglandin‐3 are derived from essential fatty acids, and are known to be therapeutically important in inflammatory conditions as well as for mental health (de Batlle et al., 2012; Huan et al., 2004; Kiecolt‐Glaser, Belury, Andridge, Malarkey, & Glaser, 2011; Peet, Brind, Ramchand, Shah, & Vankar, 2001; Perica & Delas, 2011). The omega‐6 fatty acid has been reported to have a beneficial effect on osteoporosis (Maggio et al., 2009). Ahmed, Abd, and Samad (2013) reported the therapeutic importance of omega‐3 fatty acid in protecting against bone modification in salt‐loaded rats.

Spinal cord injury leads to functional changes in autonomic function, loss of sensation, and muscle wasting (Krucoff, Rahimpour, Slutzky, Edgerton, & Turner, 2016). The induction in apoptosis in neurons and oligodendrocytes leads to axonal degeneration, demyelination, and dysfunction (Lee et al., 2003; Yune et al., 2008). In spinal cord injury, apoptosis of oligodendrocytes and neurons leads to increased oxidative stress and inflammation (Bao & Liu, 2002; Yune et al., 2008). Reactive oxygen species (ROS) and proinflammatory cytokines are produced in inflammatory and neurodegenerative states (Block & Hong, 2005; Min et al., 2004; Qin et al., 2004). Hussein, El‐Banna, Razik, and El‐Naggar (2018) reported that interleukins (IL) are essential cytokines involved in several immunological processes. The microglia release pro‐nerve growth factor (pro‐NGF), which leads to cell death (Yune et al., 2007). Therefore, novel therapeutic agents with efficacy against inflammation and oxidative stress are required. This study investigates the effects of omega‐3 fatty acids against spinal cord injury.

## 2 MATERIALS AND METHODS

### 2.1 Rats

Male albino rats were purchased from the Academy of Medical Sciences and Peking Union Medical College, Beijing, China. The rats weighed 180–210 g and were maintained in standard rat polypropylene cages and kept under standard environmental conditions of 12 hr of light and 12 hr of the dark with a relative humidity of 60% ± 5% and temperature of 25°C ± 0.5°C. All the animal experiment was carried out under the guidelines of the Department of Orthopedic Surgery, Peking Union Medical College Hospital. Ethical committees of Peking Union Medical College Hospital approved all animal care and experimental protocols. All animal surgery was conducted under anesthesia, and maximum effort was made to reduce animal suffering.

### 2.2 An experimental model of spinal cord injury

An experimental model of spinal cord injury was established in rats as described previously (Naruo et al., 2003; Sakanaka et al., 2007). Briefly, Spinal cord injury was established by longitudinal surgical incision following exposure of 1.5% halothane. The surgical incision was formed from the lower to mid thoracic vertebrae on the backside of the rats. The weight (20 g) was kept on the exposed Th12 surface for 30 min to induce injury. Then, weight was removed from the Th12 surface and muscles, and skin was sutured. Rats were kept under appropriate laboratory conditions for 24 hr.

### 2.3 Treatments

The rats were classified into four groups: group I, sham operation plus normal saline only; group II, control with spinal cord injury plus normal saline only; group III, spinal cord injury plus 50 mg/kg Omega‐3 fatty acids; and group IV, spinal cord injury plus 100 mg/kg Omega‐3 fatty acids. Normal saline was used to dissolve fatty acids. Control and sham rats were administered normal saline. Omega‐3 fatty acid solution or normal saline was administered orally to rats for 30 days.

### 2.4 Oxidative markers

Lipid peroxidation was measured as malondialdehyde (MDA) content through the measurement of thiobarbituric acid reactive species (TBARS). The final lipid peroxidation product was measured at 534 nm. Catalase, GSH, Gpx, and SOD levels were observed as described previously (Kaddour et al., 2016).

### 2.5 Inflammatory markers

Serum levels of interleukin (IL)‐6 and tumor necrosis factor‐alpha (TNF‐α) were determined as described previously (Hend, Omnia, Hekmat, & Amira, 2014). The levels of TNF‐α and IL‐6 mRNA expression were quantified as described previously (Moon et al., 2012). Following treatment, total RNA was isolated from the rat brain and converted into cDNA by oligo (dt) primers. The mRNA expression was determined by RT‐PCR. The relative ratios of mRNA expression were determined according to the 2^‐∆∆^ *^C^* ^T ^method. The primers used for mRNA amplification are shown in Table 1.

**Table 1:** List of primers used in RT‐PCR reaction for the amplification of following markers

S.No.	Markers	Forward primer	Reverse primer
1	TNF‐α	5′‐CCCAGACCCTCACACTCAGAT−3′	5′‐TTG TCC CTTGAA GAG AAC CTG−3′
2	IL−6	5′‐AAGTTTCTCTCCGCAAGATAC TTCCAGCCA−3′	5′‐AGG CAAATTTCCTGGTTATATCCA GTTT−3′
3	p53	5′‐TAACAGTTCCTGCATGGGCGGC−3′	5′‐AGGACAGGCACAAACACGCACC−3′
4	Bax	5′‐TGG AGCTGCAGAGGATGATTG−3′	5′‐GAAGTTGCCGTCAGAAAACATG−3′
5	Caspase−3	5′‐TTAATAAAGGTATCCATGGAGAACACT−3′	5′‐TTAGTGATAAAAATAGAGTTCTTTTGTGAG−3'
6	Bcl−2	5′‐CAC CCC TGG CAT CTT CTC CTT−3′	5′‐AGC GTC TTC AGA GAC AGC CAG−3′
7	proNGF	5′‐CTTCAGCATTCCCTTGACAC−3′	5′‐TGAGCACACACACGCAGGC−3′
8	GAPDH	5′‐TCCCTCAAGATTGTCAGCAA−3′	5′‐AGATCCACAACGGATACATT−3′

### 2.6 Apoptotic markers

The levels of p53, bcl‐2, bax, caspase‐3, and pro‐NGF mRNA expression were determined as described previously (Moon et al., 2012). Following treatment, total RNA was isolated from the rat brain and converted into cDNA by oligo (dt) primers. The mRNA expression was determined by RT‐PCR. The relative ratios of mRNA expression were determined according to the 2^‐∆∆^ *^C^* ^T ^method. The primers used for mRNA amplification are shown in Table 1. The protein in the animal cell homogenate was run on sodium dodecyl sulfate‐polyacrylamide gel electrophoresis (SDS‐PAGE). Polyvinylidene fluoride membrane was used for transferring the proteins. Nonspecific proteins were blocked by Tris‐buffered saline (TBST). Then, the membranes were incubated with primary and secondary antibodies, and protein levels were quantified (Moon et al., 2012).

### 2.7 Statistical analyses

Values are given as the means and standard deviation (*SD*). Analysis of variance (ANOVA) was used to analyze the data, and Turkey's *post hoc* test was carried out for comparisons. In all analyses, *p* < 0.05 was taken to indicate statistical significance.

## 3 RESULTS

In this study, the altered lipid peroxidation, GSH, SOD, Gpx, and catalase were normalized by omega‐3 fatty acid treatment (Table 2, *p* < 0.042). MDA content was enhanced by 1.14 nmol/ml in control rats. However, omega‐3 fatty acid supplementation reduced MDA content by >50% (*p* < 0.031, Table 2). In the control group, catalase, SOD, Gpx, and GSH levels were reduced by 83%, 77.6%, 74.6%, and 69.7%, respectively (Table 2, *p* < 0.023). However, omega‐3 fatty acid supplementation normalized these levels (Table 2, *p* < 0.035).

**Table 2:** Effect of omega‐3 fatty acid supplementation on oxidative markers in spinal cord injury induced rats

Markers	Group I (Sham)	Group II (Control)	Group III (50 mg/kg of omega−3 fatty acids)	Group IV (100 mg/kg of omega−3 fatty acids)
MDA (nmol/ml)	0.25 ± 0.011	1.14 ± 0.12*	0.86 ± 0.06#	0.41 ± 0.03#
Catalase (U/ml)	14.1 ± 0.6	2.4 ± 0.1*	6.3 ± 0.31#	12.1 ± 0.31#
SOD (U/ml)	319.4 ± 19.6	71.5 ± 5.8*	161.2 ± 11.4#	293.2 ± 16.1#
Gpx (U/ml)	0.63 ± 0.01	0.16 ± 0.01*	0.31 ± 0.01#	0.56 ± 0.03#
GSH (nmol/ml)	0.66 ± 0.02	0.20 ± 0.01*	0.33 ± 0.03#	0.59 ± 0.03#

* *p* < 0.05,

^#^ *p* < 0.05.

**Figure 1:**
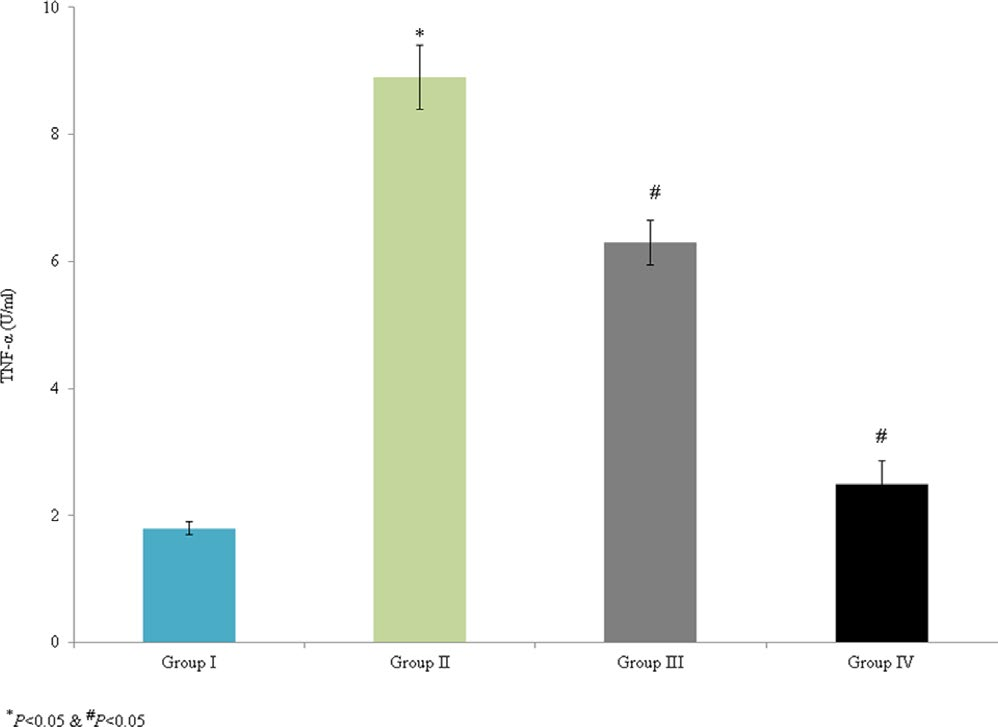
Protective effect of omega‐3 fatty acid treatment on serum TNF‐α in an experimental model of ischemia‐reperfusion‐induced rats. *p < 0.05 versus sham (group I) and ^#^ *p* < 0.05 versus control (group II, spinal cord injury plus normal saline only)

**Figure 2:**
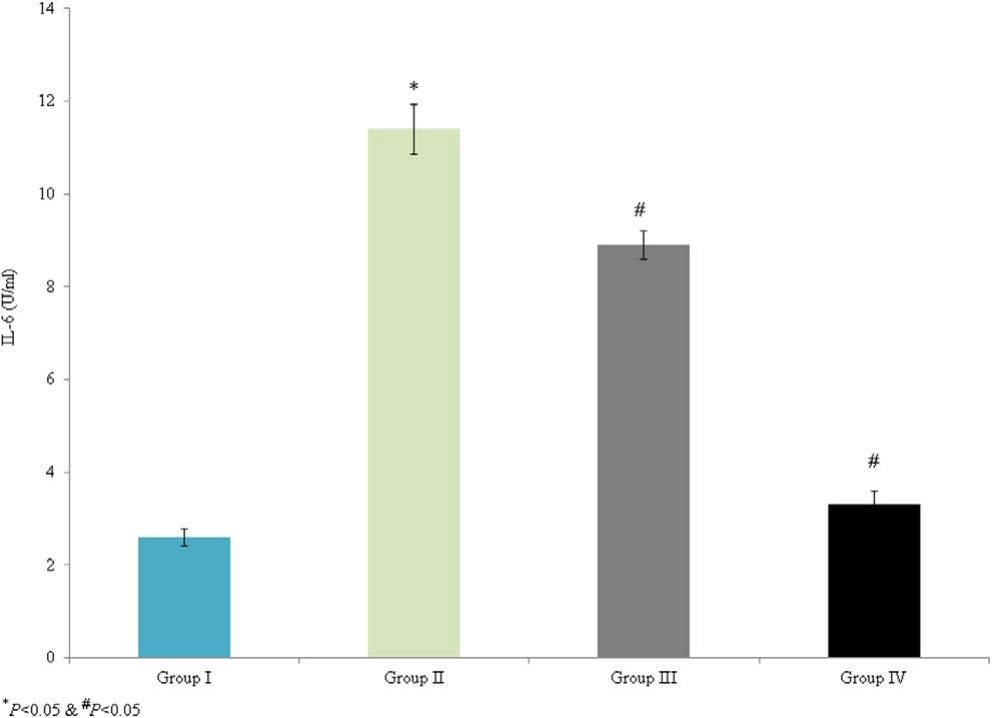
Protective effect of omega‐3 fatty acid supplementation on serum IL‐6 in an experimental model of ischemia‐reperfusion‐induced rats. *p < 0.05 versus sham (group I) and ^#^ *p* < 0.05 versus control (group II, spinal cord injury plus normal saline only)

**Figure 3:**
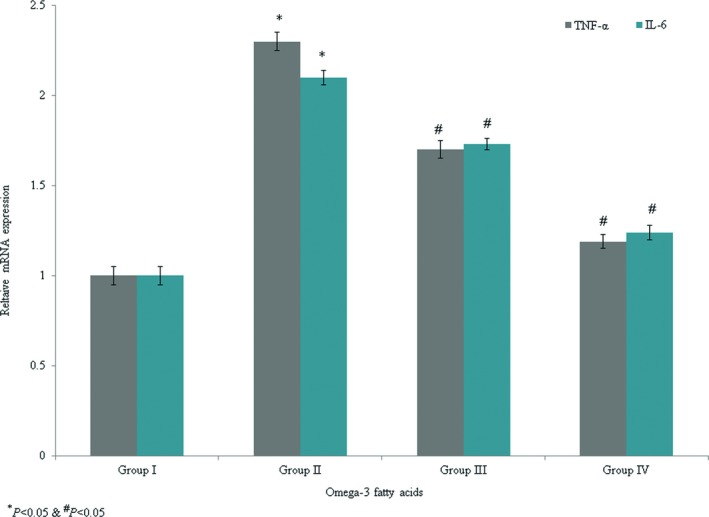
Protective efficacy of omega‐3 fatty acid supplementation on mRNA levels of TNF‐α and IL‐6 in an experimental model of ischemia‐reperfusion‐induced rats. *p < 0.05 versus sham (group I) and ^#^ *p* < 0.05 versus control (group II, spinal cord injury plus normal saline only)

The levels of TNF‐α and IL‐6 (8.9 and 11.4 U/ml, respectively) were substantially enhanced in group II. However, omega‐3 fatty acid supplementation decreased TNF‐α and IL‐6 by >50% (Figures 1 and 2, *p* < 0.042). The levels of TNF‐α and IL‐6 mRNA expression were increased by 1.3‐fold and 1.1‐fold, respectively, in the control group. However, omega‐3 fatty acid treatment significantly reduced TNF‐α mRNA expression by 0.26‐fold and 0.48‐fold in groups III and IV, respectively (Figure 3, *p* < 0.037). Omega‐3 fatty acid supplementation significantly decreased IL‐6 mRNA expression by 0.18‐fold and 0.41‐fold in groups III and IV, respectively (Figure 3, *p* < 0.046).

**Figure 4:**
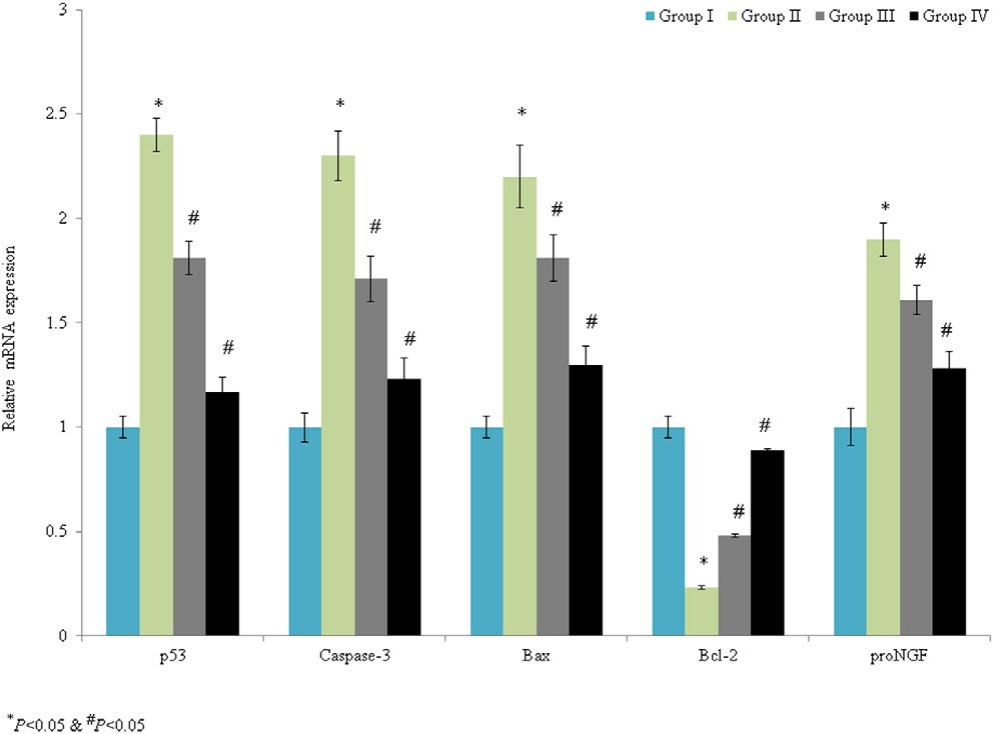
Protective effects of omega‐3 fatty acid supplementation on caspase‐3, p53, bax, bcl‐2, and pro‐NGF mRNA expression in an experimental model of ischemia‐reperfusion‐induced rats. *p < 0.05 versus sham (group I) and ^#^ *p* < 0.05 versus control (group II, spinal cord injury plus normal saline only)

**Figure 5:**
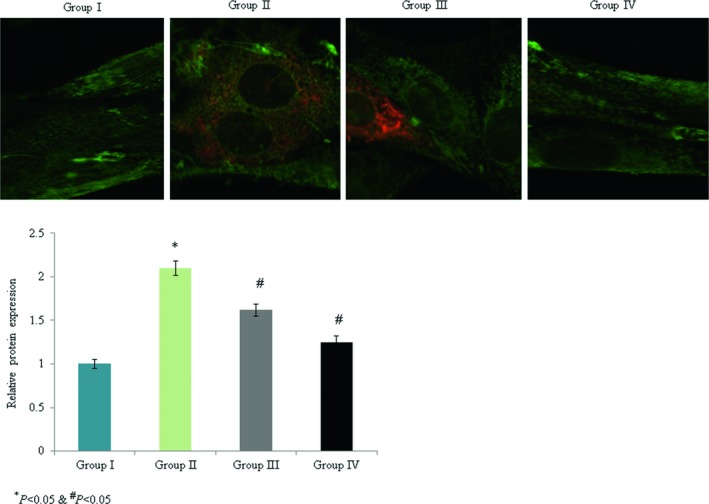
Protective effects of omega‐3 fatty acid supplementation on caspase‐3 protein expression in an experimental model of ischemia‐reperfusion‐induced rats. *p < 0.05 versus sham (group I) and ^#^ *p* < 0.05 versus control (group II, spinal cord injury plus normal saline only). Scale bar is 100 µm

**Figure 6:**
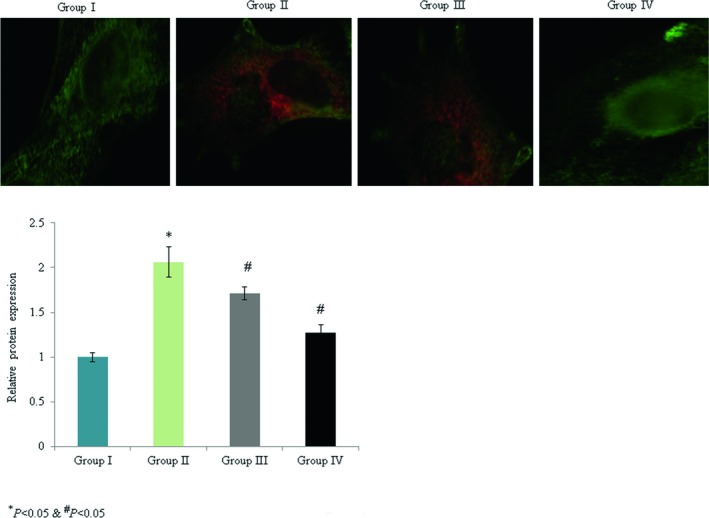
Protective effects of omega‐3 fatty acid supplementation on p53 protein expression in an experimental model of ischemia‐reperfusion‐induced rats. *p < 0.05 versus sham (group I) and ^#^ *p* < 0.05 versus control (group II, spinal cord injury plus normal saline only). Scale bar is 100 µm

The levels of p53, bcl‐2, bax, caspase‐3, and pro‐NGF mRNA expression were determined. Caspase‐3, p53, bax, and pro‐NGF mRNA expression were enhanced by 1.3‐, 1.4‐, 1.2‐, and 0.9‐fold, respectively, while bcl‐2 mRNA expression was decreased by 0.77‐fold in the control rats. Omega‐3 fatty acid supplementation significantly reduced caspase‐3, p53, bax, and pro‐NGF mRNA expression by >40%, while the level of bcl‐2 mRNA expression was enhanced by 286.9% (Figure 4, *p* < 0.033). Immunohistochemical analyses indicated that caspase‐3 and p53 protein expression levels were increased by 1.1‐fold and 1.06‐fold, respectively, in the control rats (Figures 5, 6, *p* < 0.021). Omega‐3 fatty acid treatment reduced the levels of caspase‐3 and p53 protein expression by >30% (Figures 5, 6, *p* < 0.036).

## 4 DISCUSSION

Omega‐3 fatty acids, which are essential polyunsaturated fatty acids with some roles in normal cellular metabolism (Scorletti & Byrne, 2013), have been reported to be necessary for membrane fluidity, inflammatory eicosanoids, and neural membrane oxidation (Salvati et al., 2006). Coqueiro, Bueno, and Simões (2011) reported that omega‐3 fatty acid supplementation significantly reduced acute inflammatory marker and muscle lesion marker levels. Researchers have reported the neuroprotective effect of omega‐3 polyunsaturated fatty acids against PD (Mori et al., 2018). The possible neuroprotective effect of omega‐3 fatty acids against ischemia‐induced rats (Nobre et al., 2016). Georgiou et al. (2017) have reported the neuroprotective effects of omega‐3 fatty acids against ischemic optic neuropathy induced rats. Researchers have reported the neuroprotective effect of omega‐3 fatty acids to increase neurogenesis in the culture model of hypoxic condition (Lo Van et al., 2019).

Leukotriene‐5, thromboxane‐3, and prostaglandin‐3 are derived from essential fatty acids, and are known to be therapeutically valuable in inflammation and mental health conditions (de Batlle et al., 2012; Huan et al., 2004; Kiecolt‐Glaser et al., 2011; Peet et al., 2001; Perica & Delas, 2011). Hussein et al. (2018) reported that ILs are essential cytokines involved in several immunological processes. Supplementation with omega‐3 fatty acids has been shown to reduce levels of inflammatory markers and lipid peroxidation in chronic cardiomyopathy patients (Silva et al., 2017). Hu et al. (2018) reported the beneficial effects of omega‐3 fatty acid supplementation on inflammatory markers in chronic kidney disease. Omega‐3 fatty acids reduce lipid peroxidation and increase SOD activity (Avramovic et al., 2012). The observations of reduced lipid peroxidation and elevated antioxidant marker levels observed in this study with omega‐3 fatty acid supplementation were consistent with the above findings.

Supplementation with omega‐3 fatty acid significantly inhibited oxidative stress in rat liver regeneration (Ozgur et al., 2017). Spinal cord injury leads to functional changes in autonomic function, loss of sensation, and muscle wasting (Krucoff et al., 2016). The induction in apoptosis in neurons and oligodendrocytes has been reported to lead to axonal degeneration, demyelination, and dysfunction (Lee et al., 2003; Yune et al., 2008). In spinal cord injury, apoptosis of oligodendrocytes and neurons leads to increased oxidative stress and inflammation (Bao & Liu, 2002; Yune et al., 2008). Proinflammatory cytokines and ROS are produced during inflammatory and neurodegenerative states (Block & Hong, 2005; Min et al., 2004; Qin et al., 2004). The microglia release pro‐NGF, which leads to cell death (Yune et al., 2007). Sinha et al. (2009) reported that omega‐3‐fatty acids had an antiapoptotic effect in the developing rat brain. Omega‐3 fatty acids reduced oxidative stress and apoptosis in a rat model of doxorubicin‐induced testicular damage (Uygur et al., 2014). These results were consistent with our results, indicating the inhibition of apoptosis by omega‐3 fatty acid supplementation.

## 5 CONCLUSION

In summary, the results of the study suggested that omega‐3 fatty acid supplementation significantly reduced oxidative stress, apoptosis, and the levels of inflammatory markers in ischemia‐reperfusion‐induced rats.

## DATA AVAILABILITY STATEMENT

The data that support the findings of this study are available from the corresponding author upon reasonable request.
